# Development and staging of the water flea *Daphnia magna* (Straus, 1820; Cladocera, Daphniidae) based on morphological landmarks

**DOI:** 10.1186/2041-9139-5-12

**Published:** 2014-03-18

**Authors:** Beate Mittmann, Petra Ungerer, Marleen Klann, Angelika Stollewerk, Carsten Wolff

**Affiliations:** 1 Albert-Ludwigs-Universität Freiburg, Institut für Biologie III, Neurogenetik, Lab. 5006, Schänzlestrasse 1, 79104 Freiburg, Germany; 2 Queen Mary University London, School of Biological and Chemical Sciences, Mile End Road, E1 4NS London, UK; 3 Humboldt-Universität zu Berlin, Vergleichende Zoologie, Philippstrasse 13, Haus 2, 10115 Berlin, Germany

**Keywords:** *Daphnia magna*, Embryonic development, Staging system, Morphogenesis, Cladocerans, Water flea

## Abstract

**Background:**

Crustaceans of the genus *Daphnia* are one of the oldest model organisms in ecotoxicology, ecology and evolutionary biology. The publication of the *Daphnia pulex* genome has facilitated the development of genetic tools to answer long-standing questions in these research fields (Science 331: 555-561, 2011). A particular focus is laid on understanding the genetic basis of the striking ability of daphnids to change their phenotype in response to environmental stressors. Furthermore, *Daphnia* have recently been developed into crustacean model organisms for EvoDevo research, contributing to the ongoing attempt to resolve arthropod phylogeny. These problems require the comparative analyses of gene expression and functional data, which in turn require a standardized developmental staging system for *Daphnia*.

**Results:**

Here we provide a detailed staging system of the embryonic development of *Daphnia magna* based on morphological landmarks. The staging system does not rely on developmental hours and is therefore suitable for functional and ecological experiments, which often cause developmental delays in affected embryos and thus shifts in time reference points. We provide a detailed description of each stage and include schematic drawings of all stages showing relevant morphological landmarks in order to facilitate the application of this staging scheme.

**Conclusion:**

We present here a staging system for *Daphnia magna*, which is based on morphological landmarks. The staging system can be adopted for other daphnids with minor variations since the sequence of development is highly conserved during early stages and only minor heterochronic shifts occur in late embryonic stages.

## Background

Within the large group of mainly freshwater dwelling branchiopod crustaceans, *Daphnia magna* belongs to the cladocerans, which are commonly known as ‘water fleas’. *Daphnia* is one of the oldest model organisms in ecotoxicology, ecology and evolutionary biology. The small crustacean is known for its high phenotypic adaptability reviewed by Stollewerk in [1]. The publication of the *Daphnia pulex* genome now paves the way for investigating this impressive feature on the genetic level [2]. Genetic tools, such as transgenesis and RNA interference, have become available allowing for functional analysis of eco-responsive genes, for example, see [3-6].

Furthermore, the easy cultivation and accessibility of the direct developing eggs make *D. magna* an attractive organism for evolutionary developmental studies, for example, see [7,8]. Molecular genetic studies of crustacean development are fragmentary and thus there is a large data gap that needs to be filled to understand the evolution of developmental processes in arthropods. Recent comparative studies on neurogenesis in *D. magna* have revealed considerable changes in the interactions and functions of the genes involved compared to insects [9,10]. An expansion of these studies to additional developmental processes and crustacean species is required to understand the phylogenetic relationships of insects and crustaceans and the position of Cladocera within the branchiopods.

In order to perform detailed comparative and functional studies, a staging system is therefore required. There is no staging system available for *Daphna magna*, however, several descriptions of embryonic stages have been published for various other *Daphnia* species, for example in references [5,11-15]. In most cases though, the staging is incomplete and lacks consistent morphological landmarks. Furthermore, all published staging schemes are based on the time of development after the eggs have been deposited into the brood pouch.

However, the analysis of functional and comparative data cannot rely on a staging system that is built on hours of development because treatment with chemical inhibitors, RNA interference as well as environmental stressors can lead to developmental delays. Considering the dynamic expression of developmental genes during embryogenesis, it is obvious that the informative value of functional data sets is limited if expression profiling data or *in situ* expression patterns are collected from different reference stages.

The staging system presented here for *Daphnia magna* is therefore based on morphological landmarks, which are captured by fluorescence and SEM (scanning electron microscopy). It covers the development of subitaneous eggs from the release into the brood pouch until hatching and the subsequent first instar molt.

*Daphnia magna* belongs to the Anomopoda within the Cladocera, whose embryos typically exhibit direct development as reviewed by Olesen in [16]. Anomopod crustaceans have a short body. The head is usually covered by a head shield and bears a uniramous first antenna (usually only one segment), a biramous second antenna (with the exopodite showing three to four segments, the endopodite three segments) with long swimming setae, a mandible, a first maxilla with few setae and a rudimentary or lacking second maxilla [17]. In *Daphnia* species, the thorax bears five pairs of leaf-like limbs showing great variability in shape and function and is followed posteriorly by a limbless abdomen and a telson bearing paired claws on its tip. Posterior to the head the body is covered by the carapace, which exhibits a terminal spine. During the growth season *Daphnia* reproduce asexually. The female produces parthenogenetic (apomictic/subitaneous) eggs, which are incubated in a brood pouch located dorsally underneath the carapace. The limbless abdomen can be flexed and thereby opens and closes the brood pouch. Embryos undergo direct development and hatch as not fully extended individuals (called ‘neonata’, see [18]). After being released from the mother’s brood pouch they immediately molt and look like miniaturized adults.

## Methods

### *Daphnia magna* culture and egg collection

*D. magna* (clone I1Inb1 - kindly provided by Dieter Ebert, Basel) were kept in groups of 15 to 20 individuals in any kind of glass jars (400 ml or more) filled with artificial *Daphnia* medium [19] at room temperature. They were fed with the green alga *Scenedesmus obliquus* (one stock was kindly provided by Dieter Ebert, Basel; one stock was bought from EPSAG, Göttingen) at least twice per week. The stocks of green algae were kept at room temperature in a semi-continuous culture using a 3 l Erlenmeyer flask under constant aeration and light. Algae were collected twice per week and fresh autoclaved medium was added (medium recipe after Dieter Ebert, Web guide to *Daphnia* parasites. http://evolution.unibas.ch/ebert/lab/algae.htm, Additional file 1).

*D. magna* females release their eggs into a dorsal brood pouch, which is the space between the carapace and the dorsal side of the trunk. In order to collect the eggs, females were transferred to a petri dish in a small drop of medium. While fixing them by pinning the carapace facing the petri dish down with a blunt needle, the eggs were gently removed from underneath the carapace with a second blunt needle.

### Fixation of embryos

For Hoechst 33258 (Sigma; 1 μg/ml) and SYBR® Green (Life Technologies; 1:100) nuclear staining the embryos were fixed in a 4% paraformaldehyde solution in phosphate-buffered saline (PBS) (107 mM NaOH, 136 mM NaH_2_PO_4_.H_2_O, 0.45 mM CaCl_2_, 67 mM glucose - pH 7.3) for 30 minutes. For Sytox® Green (Invitrogen; 1:1,000) nuclear staining the embryos were boiled for ten minutes in *Daphnia* medium and postfixed with 4% formaldehyde in PBS for 30 minutes.

For scanning electron microscopy embryos were fixed in Bouin’s fluid (75% saturated aqueous picrid acid solution, 20% saturated formaldehyde, 5% glacial acetic acid) for one hour. The embryos can be dechorionated and devitellinized manually using fine dissecting needles before or after fixation.

### Fluorescent nuclear stainings

Embryos were stained either with Hoechst 33258, SYBR® Green or Sytox® Green for one hour. They were washed three times for ten minutes in PBS and transferred to 70% glycerol in PBS.

### Congo-red staining

For Congo-red staining, the embryos were transferred to distilled water and the dissolved Congo-red added (1.5 mg/ml distilled water, for details see [20]). After 24 hours at room temperature, the staining process was stopped by several washes with distilled water. The embryos were subsequently transferred to 70% glycerol in PBS.

### Scanning electron microscopy

For scanning electron microscopy (SEM) the embryos were dehydrated in a graded ethanol series and dried with hexamethyldisilazane (HMDS, Roth) with one intermediate step of 1:1 ethanol 100% and HMDS. After vaporization of the HDMS the specimens were mounted and sputtered with gold using an Agar Auto Sputter Coater. A SEM FEI Inspect F was used to take the images.

### Data analysis

A Leica DM IL FLUO with Leica DFC420C camera was used for documentation. Confocal laser-scanning data stacks were obtained using a Leica SP5 microscope or a Leica SP2 microscope, respectively. The confocal image stacks were analyzed with the 3D-reconstruction software IMARIS (Bitplane AG, Switzerland); volume renderings were created when needed.

## Results

### S1: Egg cell (apomictic)

**Figure 1:**
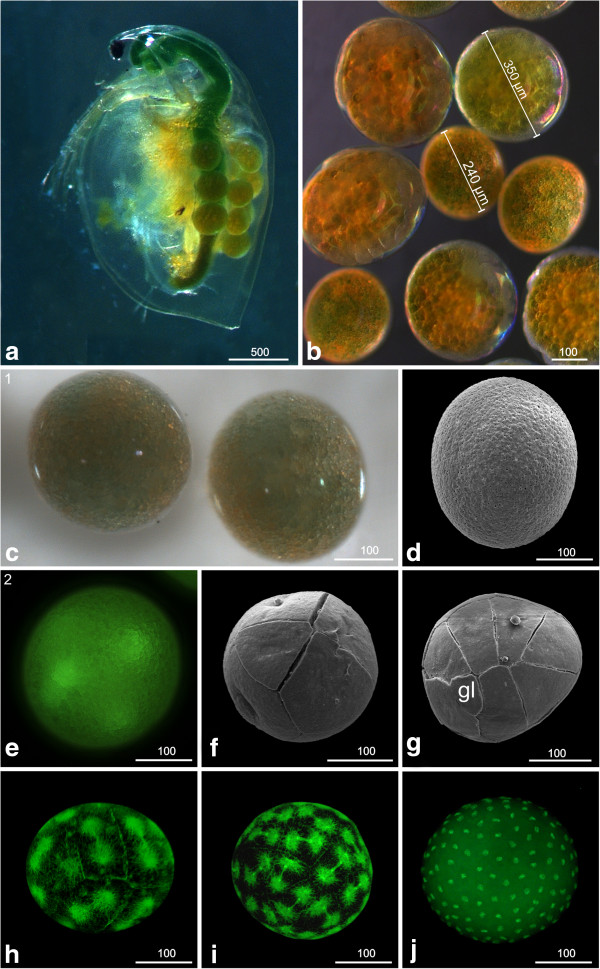
**Early developmental stages (stage 1: Egg cell (apomictic), (a-d); stage 2: Early cleavages, (e-j): a, b, c light microscopy; d, f, g SEM; e, h, i, j Sytox. (a)** Female of *D. magna* carrying eggs in its brood pouch. The size of the eggs can vary even in one single female. **(b)** Eggs of several females. The size can vary by more than 60%. **(c)** Stage 1 (egg cell; apomictic); the nucleus lies within non-transparent globular pale to greenish yolk granules and a number of larger oil drops. **(d)** The egg is covered tightly by the chorion. **(e)** Stage 2 (Early cleavages); the first cleavages of the egg are intralecithal. **(f)** From the eight-nuclei stage onward, cell membranes are visible externally (arrowhead) and demarcate the blastomeres. **(g)** The 16-nuclei stage exhibits a characteristic arrangement of blastomeres which are unequal in size. One small cell indicated by a ‘gl’ could be the germ line precursor cell. **(h)** 16 to 32 cells. **(i)** 32 to 64 cells. **(j)** More than 512 cells. The first nine cleavage cycles are synchronous. gl = germ line.

*Daphnia magna* subitaneous egg cells are released into the brood pouch from the ovaries; they vary considerably in size between clutches of different mothers. The size of the eggs is between approximately 240 and 350 μm; differences in size can be more than 60% (Figure 1a, b). Eggs are more or less spherical shaped and are covered tightly by a transparent egg membrane, the chorion (Figure 1c, d). It is possible that the vitelline membrane is present underneath the chorion but this could not be verified with the methods used here. The egg nucleus lies within non-transparent globular greenish yolk granules and a number of larger oil drops (Figure 1c). The oil drops seem to move deeper inside the yolk after onset of the first cleavage divisions.

### S2: Early cleavages

The first cleavages are intralecithal (Figure 1e; see also [21]). Membranes separating the individual blastomeres are not visible from the outside until the eight-nuclei stage (Figure 1f). (Please note that the techniques applied here do not allow for determining the starting point of complete cytokinesis; however, to maintain consistency we use the term ‘blastomere’ throughout.)

The early blastomeres are unequal in size so that a characteristic arrangement can be observed. In the 16-nuclei stage, for example, a single noticeably smaller cell is located at one pole of the egg (marked with gl in Figure 1g), which might develop into a germ line precursor cell [21,22].

Continuing synchronous cleavage cycles (n = 9) result in a blastoderm showing about 512 cells at late stage 2 (Figure 1h-j). We could not detect any blastoderm cells inside the yolk mass.

### S3: Gastrulation zone

**Figure 2:**
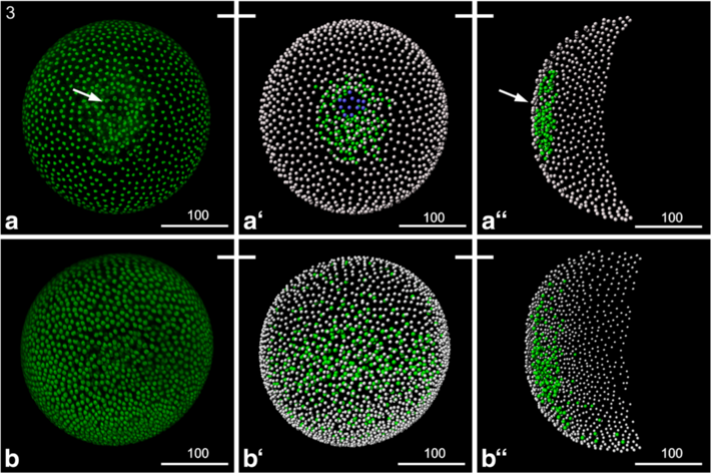
**Stage 3 (Gastrulation zone): (a, b) Sytox; (a‘, a“, b‘, b“) Volume rendering.** A white bar connecting two pictures point out that the same individual is shown. **(a)** The ring like gastrulation zone (arrow) is clearly visible. Also the larger cells, probably the germ cells, are clearly visible. **(a‘)** The Volume rendering shows the ectodermal cells (grey), the mesendodermal cells (green) and the larger potential germ cells (blue). **(a“)** Transversal view of the same construct shows that the internalized cells just start to migrate. **(b)** Later on the gastrulation zone starts to disappear and the internalized cells spread underneath the ectodermal cell layer. **(b‘)** The Volume rendering shows the ectodermal cells (grey) and the mesendodermal cells (green) continuing their migration. **(b“)** Transverse view of the same construct shows that the internalized cells migrated off the gastrulation zone; they spread in a bilaterally symmetrical manner more extensively towards the posterior and lateral than towards the anterior, leading to the disappearance of the gastrulation zone.

Gastrulation occurs by immigration of cells at a confined gastrulation zone and subsequent epiboly (arrow in Figure 2a). At this stage, there seems to be a higher concentration of nuclei in one pole of the egg (the future ventral side) with the gastrulation zone in the center of this dense area.

Shortly after the first cells are detectable inside, noticeably larger cells appear in the center of the gastrulation zone (blue cells in Figure 2a‘). These large cells eventually immigrate as well, followed by additional smaller cells. According to observations in *Daphnia pulex* the large cells correspond to the germ line cells [21,23]. It seems that the internalized cells spread more extensively towards posterior than towards anterior (Figure 2a). Further epibolic movements result in bilaterally symmetrical spreading of the internalized cells along the dorso-ventral axis and by the end of this stage the gastrulation zone is not longer detectable externally (Figure 2b).

### S4: Appearance of the ‘Scheitelplatten’

**Figure 3:**
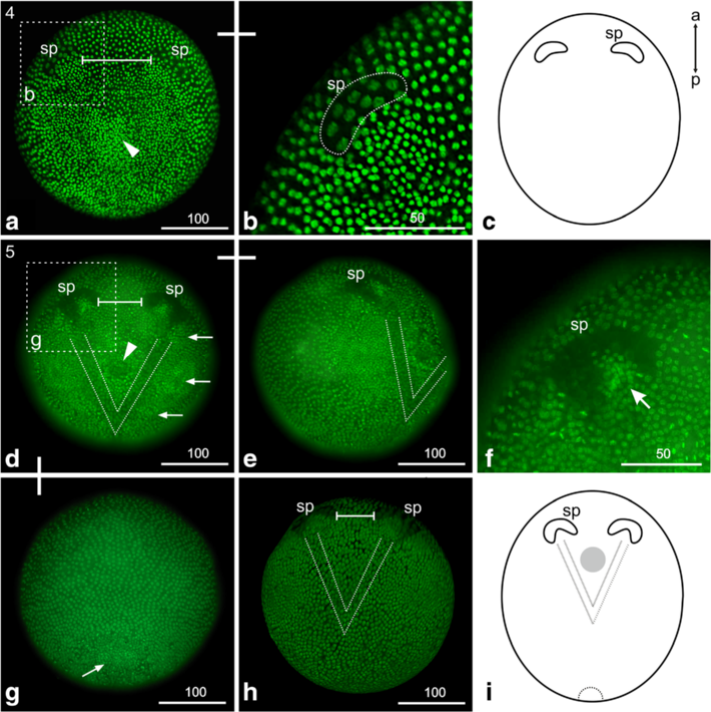
**Stage 4 (Appearance of the ‘Scheitelplatten’, (a-c)) and stage 5 (Head-V, (d-i)): (a, b) Sytox; (c, i) Schematic drawing; (d-h) Hoechst. (a)** Stage 4 (appearance of the ‘Scheitelplatten’); a paired ‘Scheitelplatten’ anlage appears anterior to the gastrulation zone. Each ‘Scheitelplatte’ consists of a cluster of about ten to twelve cells; this number increases during further development. The bar marks the distance between the pair of ‘Scheitelplatten’. The arrow marks a field of densely packed cells, which is the later mouth field. **(b)** Detail of one kidney-shaped ‘Scheitelplatte’ which becomes more crescent-shaped during further development. **(c)** Schematic drawing of an early stage 4embryo. **(d)** Stage 5 (Head-V); in the head region a V-shaped area becomes apparent. Its posterior tip extends to the prospective posterior border of the mandibular segment. The cells show loosely scattered nuclei resulting in a lower fluorescent nuclei staining compared to the adjacent area. The bar marks the decreased distance between the ‘Scheitelplatten’. The arrows mark the regions of the emerging naupliar appendages which are the first and second antennae and the mandible. The arrowhead marks the area of the later stomodeum. **(e)** Lateral view of the same embryo. **(f)** Detail of the ‘Scheitelplatte’, which shows a crescent-shape, enclosing clusters of small cells with bright nuclear staining (arrow). **(g)** At the posterior end the area of the prospective proctodeum is evident due to a cluster of internal cells and an external roundish ‘flap’-like incision (arrow). **(h)** The ‘Scheitelplatten’ move even closer together during further development. **(i)** Schematic drawing of the Head-V stage. sp = ‘Scheitelplatte’.

A conspicuous paired structure - called ‘Scheitelplatte’, for example, [22,24] appears anterior to the gastrulation zone (Figure 3a). Initially, each ‘Scheitelplatte’ consists of a cluster of about ten to twelve large cells, which together form a kidney-shaped area (Figure 3b). The nuclei of these cells are less condensed and therefore show a lower signal when stained with fluorescent nuclear markers (that is Hoechst, Sytox) compared to the surrounding cells (Figure 3a, b). The ‘Scheitelplatten’ are the first morphological landmarks that indicate the position of the anterior-posterior as well as the dorso-ventral axis (see text below). In the region of the former gastrulation area a large number of internal mesendodermal cells are visible (arrowhead in Figure 3a). Furthermore, the ectodermal cells overlying this area are smaller and more densely packed. During further development the ‘Scheitelplatten’ consist of more cells, leading to a crescent-like shape (compare Figure 3a-c and d, f).

### S5: Head V

A V-shaped area demarcates the field where the anterior part of the nervous system forms, including the brain. The area is characterized by loosely arranged nuclei, which results in an overall lower staining compared to the surrounding cells, and fewer cells underneath the ectoderm compared to the adjacent area (dotted lines in Figure 3d, e, h, i). The V-shaped area is positioned directly posterior to the ‘Scheitelplatten’ and its posterior tip extends to the prospective posterior border of the mandibular segment. We stained embryos with *Dam snail* a neural marker, which labels neuroblasts and their precursors [9]. The ‘Scheitelplatten’, as well as the V-shaped area strongly express the marker indicating that these structures form the neural tissue of the naupliar region (See Additional file 2).

Lateral to the V, areas of condensed cells mark the regions of the emerging naupliar appendages (first and second antennae and mandible, arrows in Figure 3d). The arising stomodeal invagination, which is located in the center of the V, is foreshadowed by a circular area of low fluorescent signal (arrow head in Figure 3d).

The crescent-shaped ‘Scheitelplatten’ are located closer to each other than in the previous stage (compare Figure 3a and d). The nuclei of the ‘Scheitelplatten’ cells are located basally and continue to show lower fluorescent staining than the surrounding cells (Figure 3f). Each ‘Scheitelplatte’ encloses a posteriorly abutting cluster of small cells, which shows bright fluorescent nuclear staining (compare Figure 3a and d). It has been suggested that the ‘Scheitelplatten’ contribute to the visual system among others in Cladocera [24] and we therefore analyzed the expression of *atonal*, a gene showing a conserved function in eye development throughout the animal kingdom. In line with the assumption, the ‘Scheitelplatten’ are associated with an anterior cluster of *atonal* positive cells (Additional file 2).

At the posterior pole, the prospective proctodeum is evident due to a cluster of internal cells and an external roundish ‘flap’-like incision (arrow in Figure 3g). Otherwise, only one cell layer beneath the ectoderm in the dorsal trunk region is present at this stage.

### S6: Naupliar segments

During this stage, the anlagen of the naupliar appendages appear in the following order: second antenna, first antenna and mandible. According to these landmarks, S6 can be divided into two sub-stages.

#### *Second antenna*

**Figure 4:**
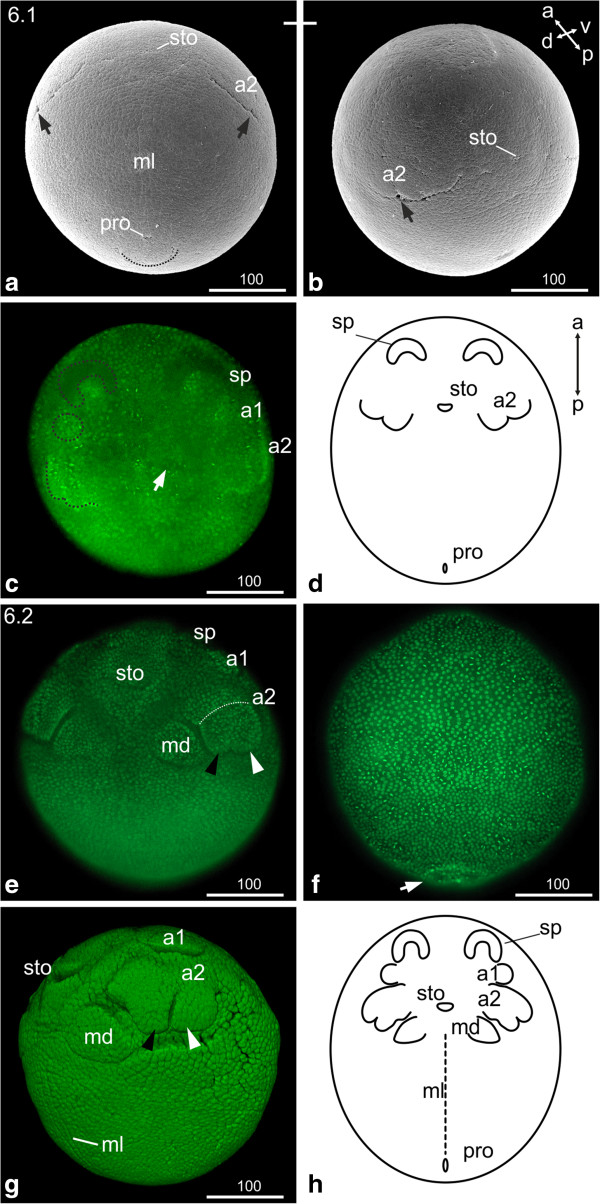
**Stage 6 (Naupliar segments, 6.1 (a-d); 6.2 (e-h)): (a, b) SEM; (c, e, f, g) Sytox; (d, h) Schematic drawing. (a)** Stage 6.1; Ventral view: the second antennae appear as undulated transverse furrow; the arrow indicates the indentation where endo- and exopodite will form. The stomodeum appears as transverse slit. The midline cells of the developing neuroectoderm are evident. The proctodeum is visible as a ‘pore’ anterior to a ‘flap’-like structure (dashed line). **(b)** The same embryo as in (a) from a different view. **(c)** Ventral view: an area of higher nuclei density announces the appearance of the first antennae (dashed circle). The arrow points to the stomodeal area. **(d)** Schematic drawing of stage 6.1. **(e)** Stage 6.2, ventral view: the endo- and exopodal lobes of the second antennae elongate. The dashed line indicates the partition of the most proximal segment. The black arrowhead indicates the endopodite; the white arrowhead indicates the exopodite. The buds of the first antennae are evident. Mandible buds have formed medially to the second antennae. **(f)** Dorsal view: the ‘flap’-like incision of the proctodeal area is evident (arrow). **(g)** Lateral view: the buds of the first antennae are evident. The subdivision of the second antennae in to basal segment, endopodite (black arrowhead) and exopodite (white arrowhead) become clearer. The stomodeum appears as transverse slit giving the embryo a smiling appearance. The midline cells can be distinguished as longitudinal line reaching from the posterior edge of the mandibular segment to the proctodeum; they are separated via longitudinal indentations from the lateral neuroectoderm. **(h)** Schematic drawing of stage 6.2. a1, a2 = first and second antennae; md = mandible; ml = midline; pro = proctodeum; sp = ‘Scheitelplatte’; sto = stomodeum.

The first visible signs of the second antenna appear as undulated transverse furrows with one indentation indicating where the endo- and exopodite will form (arrows in Figure 4a, b). Areas of higher nuclei density demarcate the origin of the first antennae (Figure 4c). The stomodeum is evident as a narrow transverse slit at the level of the anlagen of the second antennae. A longitudinal ectodermal midline cell row becomes separated from the lateral neuroectoderm which is indicated by the regular alignment of the respective cells as well as a longitudinal indentation extending from the posterior area of the maxillae to the proctodeal area (ml in Figure 4a). The proctodeum is visible as a ‘pore’ anterior to a ‘flap’-like structure (Figure 4a, d). The second membrane, the vitelline membrane, covers the embryo tightly beneath the chorion.

#### *First antenna and mandible*

The buds of the first antennae are visible posterior to the lateral border of the ‘Scheitelplatten’; the distal part of the buds points laterally (Figure 4e, h). The attachment sites of the first and second antennae are clearly separated at this stage.

The endo- and exopodal lobes of the second antennae are elongated and the most proximal segment is visible (dotted line in Figure 4e). Large mandible buds have formed postero-medially to the second antennae, with the distal part pointing laterally (Figure 4e, g, h). The invaginating stomodeum appears as wide transverse slit. Bilateral longitudinal indentations clearly separate the midline region from the lateral neuroectoderm. The midline cells become more distinct by broadening along the medio-lateral axis (ml in Figure 4g). The proctodeum is externally visible as a longitudinal slit inside the ‘flap’-like incision (just the ‘flap’-like incision is shown in Figure 4f).

### S7: Postnaupliar segments

#### *First thoracic segment*

**Figure 5:**
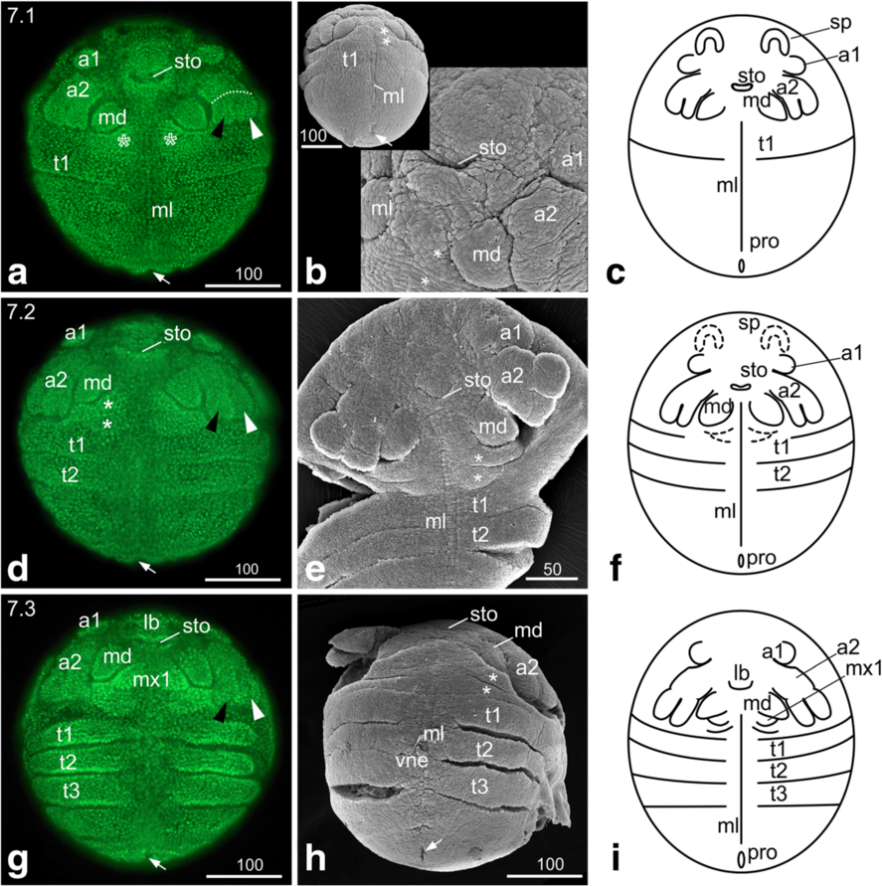
**Stage 7 (Postnaupliar segments, 7.1 (a-c); 7.2 (d-f); 7.3 (g-i)). (a, d, g) Sytox; (b, e, h) SEM; (c, f, i) Schematic drawing. (a)** Stage 7.1, ventral view: the tips of the second antennae and the mandibles are arranged in a horizontal line. The asterisks mark the maxillary zone. The arrow marks the area of the proctodeum. **(b)** The insertions of the first and second antennae have come to lie next to each other. The ‘flap’-like structure posterior of the proctodeum (arrow) disappeared. **(c)** Schematic drawing of stage 7.1. **(d)** Stage 7.2, ventral view: The first antennae have moved medially. The second antennae are elongated, and the tips of their exo- and endopodites delimit the maxillary zone laterally. The asterisks mark the segments of the first and second maxillae. The second thoracic segment is separated from the rest of the trunk by an intersegmental furrow except for the area of the neuroectoderm. **(e)** Ventral view: the SEM of stage 7.2 provides a clear view of the intersegmental furrows between the first and second maxillae (asterisks), between the first and second thoracopod and the rest of the trunk as well as of the longitudinal midline of the neuroectoderm. **(f)** Schematic drawing of stage 7.2. **(g)** Stage 7.3. Ventral view: the second antennae have continued elongation and almost extend to the first thoracic segment. The mandibles have increased in size, and the emerging first maxillae become evident. The third thoracic segment has formed. The limb anlagen of the thoracic segments become evident by uneven boarders; in late stage 7.3 embryos slight elevations can be detected. **(h)** The prolonged second antennae and the enlarged mandibles are evident. Also the undulated boarders of the first, second and third thoracopod due to the developing limb buds are obvious. The lateral ventral neuroectoderm can be distinguished from the midline cells by its uneven surface, which seems to be related to the development of neuroblasts. **(i)** Schematic drawing of stage 7.3. a1, a2 = first and second antennae; lb = labrum; md = mandible; ml = midline; mx1, mx2 = first and second maxillae; ne = neuroectoderm; pro = proctodeum; sp = ‘Scheitelplatte’; sto = stomodeum; t = thoracic segment; vne = ventral neuroectoderm.

The attachment sites of the first and second antennae have come to lie next to each other (Figure 5a, b). The tips of the endo- and exopodite of the second antennae and the tips of the mandibles are arranged in a horizontal line (Figure 5a). The region where the first and second maxillae form - called the maxillary zone [13] - is demarcated by condensed cells showing a strong fluorescent nuclear signal (asterisks in Figure 5a). The first intersegmental furrow in the trunk outlines the posterior border of the first thoracic segment (Figure 5a, c). The intersegmental furrow is not continuous but interrupted in the ventro-medial area which gives rise to the midline and ventral neuroectoderm. This applies to all subsequently forming intersegmental furrows (Figure 5a, d-i).

The midline cells are unchanged compared to stage 6. The ‘flap’-like structure posterior of the slit-like proctodeum has disappeared (arrow in Figure 5b).

#### *Second thoracic segment*

The first antennae are positioned more medially compared to stage 7.1 The ‘Scheitelplatten’ have continued to shift medially; their crescent-like form has partly dissolved and they are less noticeable. The second antennae have elongated further; the tips of their endo- and exopodites delimit the maxillary zone laterally (Figure 5d-f). The segments of the first and second maxillae are separated by a segmental furrow (asterisks in Figure 5d, e). The segmental furrow between the second and third thoracic segment is also evident (Figure 5d-f).

#### *Third thoracic segment*

The second antennae have further elongated and almost extend to the first thoracic segment. The size of the mandibles has increased and the outlines of the emerging first maxillae can be visualized by fluorescent nuclear staining (Figure 5g). The third thoracic segment has formed. The segmental borders start to become uneven in the thoracic segments due to the formation of the limb anlagen, which appear as slight elevations in late stage 7.3 embryos (Figure 5g, h). The lateral (distal) border of the limb buds is not yet visible (Figure 5i). At this stage the first morphological manifestation of neurogenesis can be observed. Most neuroblasts (neural stem cells) are generated at this stage [9,10] and the ventral neuroectoderm can now clearly be distinguished from the ventral midline and the lateral limb anlagen (Figure 5h).

#### *Fourth thoracic segment*

**Figure 6:**
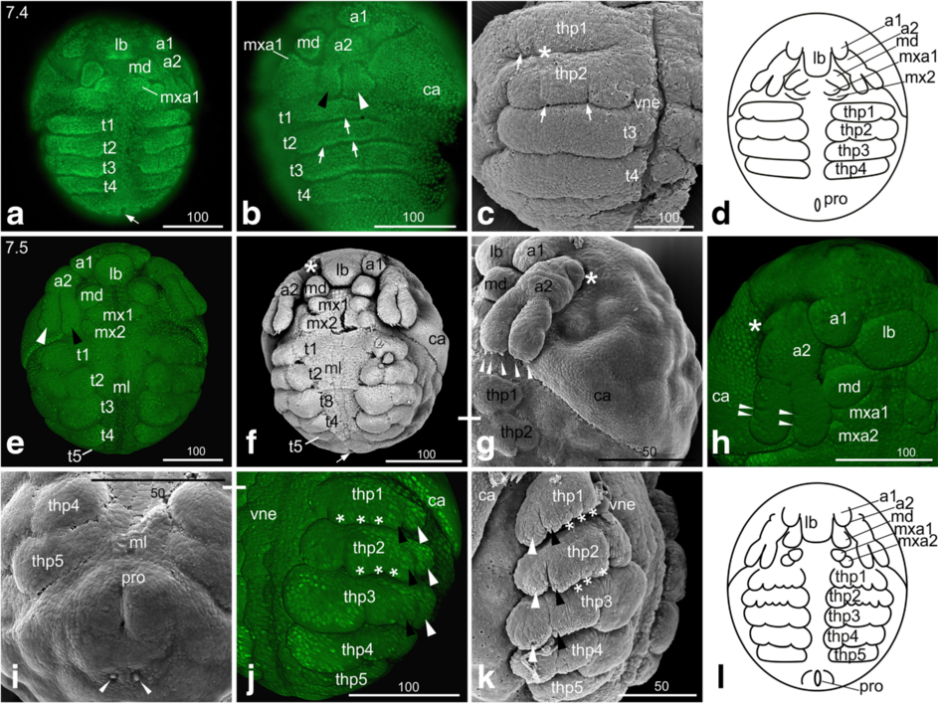
**Stage 7 (Postnaupliar segments; 7.4 (a-d); 7.5 (e-l)): (a, b, e, h, j) Sytox; (c, f, g, i, k) SEM; (d, l) Schematic drawing. (a)** Stage 7.4, ventral view: The first antennae are located closer to the labrum anlage, which appears as a roundish protrusion and covers the stomodeum. The arrow points to the proctodeum. **(b)** Lateral view: The differentiation of the first and second thoracopods is indicated by slight indents (arrows). The first thoracopod shows one indentation, which marks the subdivision into later exo- and endopodite (arrow), second thoracopod shows two indentations (arrows) dividing the limb bud into one basal and two distal segments. The black arrowhead indicates the endopodite; the white arrowhead indicates the exopodite of the second antennae. **(c)** Ventro-lateral view: the SEM shows clearly the indentation of the first and second thoracopod (arrows); the depression medially to the indentation (asterisk) of first thoracopod is an artefact from mounting the embryo. **(d)** Schematic drawing of stage 7.4. **(e, f)** Stage 7.5, ventral view: the insertion of the first antennae abuts the insertion of the slightly more rectangular labrum. The first maxillary bud is positioned in a longitudinal line with the mandible, whereas the second maxilla lies slightly more laterally. The asterisk marks an artifact as a1 was lost during mounting. The arrow points to the proctodeum. **(g)** The tips of the endopodite of the second antenna shows three setae-buds, the exopodite of the second antenna shows two setae-buds (arrow heads). In addition, one bud of a developing seta is evident at the medial side of the endopodite approximately half way through its length. **(h)** The endo- and exopodite of the second antenna show each two slight indentations (arrowheads), which indicate the final three-partite segmentation. At the proximal segment of the second antenna an outgrowth becomes evident postero-laterally (asterisk). **(i)** The area around the slit-like proctodeum becomes bulky. The arrowheads point to a pair of setae posterior to the proctodeum. **(j, k)** The thoracopods show their continuing differentiation with a developmental gradient from anterior to posterior. **(l)** Schematic drawing of stage 7.5. a1, a2 = first and second antennae; ca = carapace; lb = labrum; md = mandible; ml = midline; mx1, mx2 = first and second maxillae; pro = proctodeum; t = thoracic segment; thp = thoracopod; vne = ventral neuroectoderm.

The first antennae are located more medially than in the previous stage, closer to the labrum anlage, which appears as an unpaired, roundish protrusion covering the stomodeum (Figure 6a). Besides a slight elongation, the second antennae have not changed remarkably compared to the previous sub-stage. The buds of the first maxillae appear medially (Figure 6a).

Although the limb buds are still not demarcated laterally, the differentiation of the limb segments is indicated by slight indents on the prospective first and second thoracopods (Figure 6b, c). Only one indentation occurs laterally at the first thoracopod, which divides the limb bud into the later exopodite and endopodite (arrow in Figure 6b, c). In the second thoracopod, two indentations are visible in the area where the thoracopod becomes separated into a proximal and two distal segments, the endopodite and the exopodite (arrows in Figure 6b, c). The fourth thoracic segment is thus formed (Figure 6a-d).

The carapace anlage is visible in the dorso-anterior region of the embryo as a cell sheet showing higher density than the rest of the dorsal side of the embryo. An indentation extending from the lateral area of the maxillary segments towards dorsal demarcates the outline of the carapace anlage (Figure 6b).

The midline is unchanged showing one distinct cell row, which can clearly be distinguished from the adjacent cells of the neuroectoderm.

#### *Fifth thoracic segment*

The first antennae have assumed a roundish shape and their insertions abut the slightly more rectangular labrum anlage medially and the second antennae laterally (Figure 6e-h). The second antennae have further elongated but still do not reach the first thoracic segment as the entire head region has enlarged (Figure 6e, f, g). The endo- and exopodite of the second antenna show each two slight indentations indicating the final three-partite segmentation (arrowheads in Figure 6h). At the tips of the endo- and exopodite, buds of developing setae become evident: the endopodite shows three setae-buds, the exopodite one or two (Figure 6g). In addition, one bud of a developing seta is evident at the medial side of the endopodite approximately half way through its length (Figure 6f, not separately marked). In late stage 7.5 embryos, an additional seta-bud appears more proximally (data not shown). At the proximal segment of the second antenna an outgrowth becomes evident (this tiny outgrowth seems to appear earlier but is difficult to spot) posterior-laterally (asterisk in Figure 6g, h).

The mandibles are visible as prominent roundish buds anterior to the first and second maxillae, which also appear as roundish buds (Figure 6e, f, h). The first maxilla is positioned in a longitudinal line with the mandible, whereas the second maxilla lies slightly more laterally (Figure 6e, f, h).

The fifth thoracic segment appears and immediately forms limb buds (Figure 6f, i, j, k). All thoracopods are laterally demarcated by indentations and all of them besides the fifth thoracopod continue forming distinct lobes (Figure 6j, k), in particular the second and third thoracopods. Two indentations subdivide the first thoracopod into a small basal segment, an endopodite and an exopodite (Figure 6j, k); the endopodite shows three distinct endites (asterisks in Figure 6j, k). In the second thoracopod, the subdivision into basal segment, endopodite and exopodite as well as the three endites become more distinct (Figure 6j, k). The third and fourth thoracopods also show the three-partite subdivision, but endites have not formed yet. The bud of the fifth thoracopod appears more or less rectangular (Figure 6f, i-l).

The carapace anlage covers the embryo on its dorsal side like a cape, which originates ventro-medially from the lateral sides of the second maxillary segment. It covers approximately half of the dorsal area of the embryo. Its posterior rim appears slightly wedge-shaped (Figure 6e, f, g, j). Antero-dorsally, an oval field of less brightly stained cells indicates the dorsal organ (data not shown).

The midline and the neuroectoderm are even more distinct showing clear lateral and medial boundaries (Figure 6e, f, i, j). The area around the slit-like proctodeum becomes bulky. Posterior to the proctodeum a pair of setae is detectable (Figure 6i).

At this stage the embryo sheds off the chorion and starts to extend along the anterior-posterior axis.

### S8: First antennae partially overlapping the labrum

**Figure 7:**
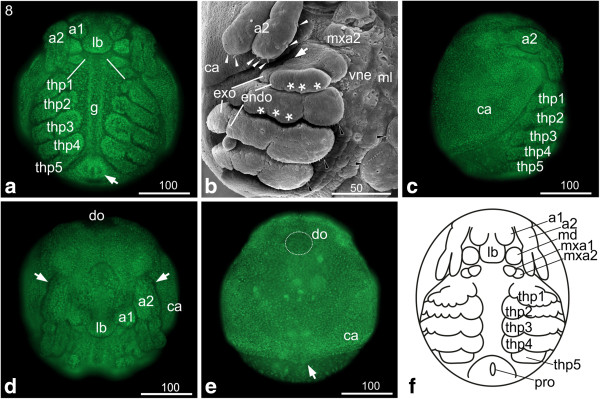
**Stage 8 (First antenna partially overlapping the labrum): (a, c, d, e) Sytox; (b) SEM; (f) Schematic drawing. (a)** Ventral view: the flattened first antennae have continued to shift medially and partly overlap the labrum. The second antennae have elongated and reach the first thoracopod. The buds of the second maxillae have moved laterally causing the buds of maxillary appendages being lined up at a 45 degree angle relative to the anterior-posterior axis (white lines). Underneath the midline the developing gut becomes distinct. The proctodeum (arrow) has started to shift anteriorly; therefore it is positioned more medially to the fifth thoracopods. **(b)** Ventro-lateral view: endo- and exopodite of the second antenna show a regular arrangement of setae-buds (white arrow heads) on the tips and on the medial rim of the endopodite. The endo- and exopodites of the first to fourth thoracopod became more distinct. Tiny setae pointing to the medial food groove are visible at the margin of the basal segment of the second to fourth thoracopod (black arrowheads). **(c)** The lateral view shows the cape-like carapace. **(d)** The dorso-frontal view shows the first antenna overlapping the elongating labrum. The arrow marks the bud at the proximal segment of the first antenna. **(e)** Dorsal view: the carapace covers more than two thirds of the posterior area of the embryo. At the posterior edge it shows a slight medial bulge pointing posterior (arrow). The dorsal organ (highlighted by dotted line) shows a spherical shape. **(f)** Schematic drawing of stage 8. a1, a2 = first and second antennae; ca = carapace; g = gut; gi = gill; lb = labrum; md = mandible; mxa = maxillary appendage; pro = proctodeum; thp = thoracopod; vne = ventral neuroectoderm.

The buds of the first antennae have flattened and shifted more medially compared to the previous stage so that they now partly overlap the labrum (Figure 7a). The second antennae have further elongated and become long and slim; they now extend to the first thoracic segment (Figure 7a, b). At the lateral edge of the endopodite, two more buds of sensillae are evident in the distal area; the proximal one is located at the level of the indentation, the other one more distally (Figure 7b). The buds of the second maxillae have moved slightly laterally; due to this movement the lined up first and second maxillae are positioned at a 45 degree angle relative to the anterior-posterior axis (white lines in Figure 7a). The indentation between endo- and exopodite in the first to fourth thoracopods is extended (Figure 7b). However, the overall-shape does not change remarkably. The fifth thoracopod still appears as a more or less rectangular bud (Figure 7a, c). Furthermore, the gills start to grow out laterally from the basal segment of the thoracopods (white arrow in Figure 7b). Tiny setae are visible at the posterior margin of the basal segment of the second to fourth thoracopods (black arrowheads in Figure 7b). The relative position of the fifth thoracopod and the proctodeum has changed. The proctodeum, which is located at the posterior tip of the abdomen, starts to shift anteriorly resulting in the proctodeum being positioned more medially to the fifth thoracopods compared to the previous stage (Figure 7a, f).

Underneath the midline the developing gut becomes distinct (g in Figure 7a). The carapace covers more than two thirds of the dorsal area of the embryo. It shows a slight medial bulge at its posterior edge, which points posteriorly (arrow in Figure 7e). The dorsal organ assumes a spherical shape (Figure 7d, e). Beneath the vitelline membrane, a putative transparent embryonic cuticle starts to form (data not shown).

### S9: First and second maxillae in horizontal line

**Figure 8:**
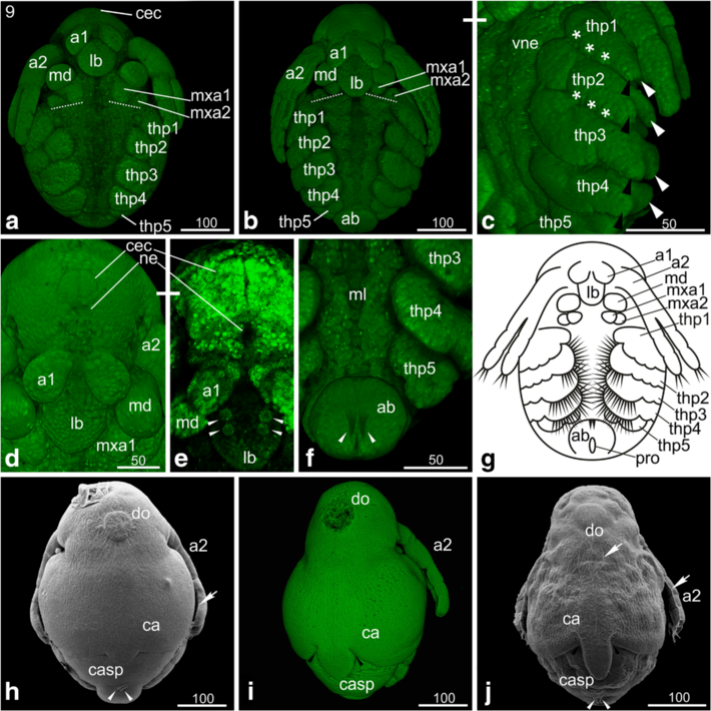
**Stage 9 (first and second maxillae in horizontal line): (a-f, i) Sytox; (g) Schematic drawing; (h, j) SEM. (a, b)** Ventral view: the first antennae have shifted further medially, their insertions touching medially at the end of this stage. The second maxillae have moved further laterally so that they form a horizontal line with the first maxillae (dashed lines). **(c)** Ventro-lateral view of the thoracopods: first to fourth thoracopod continue differentiation which results in elongation of endopodites (black arrow head) and exopodites (white arrow heads) pointing postero-laterally. **(d, e)** In front of the compound eye anlage the nauplius eye is evident which appears in living animals as brownish eye pigment spot. The arrowheads point at the four maxillary glands in the labrum. **(f)** The fifth thoracopod lost its rectangular shape and shows one indentation; no setae are evident. The outgrowth of the abdomen leads to a further protruding of the proctodeum. The arrowheads indicate two developing abdominal furcal claws, which point in posterior direction. **(g)** Schematic drawing of stage 9. **(h, i, j)** The carapace protrusion narrows, becoming a short triangular process and finally a broad elongated spine. The black arrowheads in **(i)** and **(j)** point to the area where tissue movement takes place and indentations occur leading to a change in the overall-shape of the carapace. The white arrows point to the wrinkles of the embryonic cuticle. The white arrowheads in **(h)** and **(j)** point to the elongated two setae posterior of the proctodeum. **(i)** Shows that the dorsal organ contains only a few cells. a1, a2 = first and second antennae; ab = abdomen; ca = carapace; cec = compound eye capsule; casp = carapace spine; do = dorsal organ; lb = labrum; md = mandible; ml = midline; mxa = maxillary appendage; ne = neuroectoderm; pro = proctodeum; thp = thoracopod; vne = ventral neuroectoderm.

The main character of stage 9 is the movement of the second maxillae resulting in the first and second maxillae being positioned in an almost horizontal line by the end of this stage (see dashed lines in Figure 8a, b). In addition, we observe a continuous medial movement of the first antennae towards each other, so that by the end of this stage their bases touch or even appear fused (Figure 8a, b, d, e). However, this landmark is not reliable as it either shows heterochrony or the extent of the basal fusion is sex specific.

The labrum has further elongated and the labral glands are prominent structures in fluorescent nuclear staining (arrowheads in Figure 8e). The faintly colored paired anlage of the compound eye/compound eye capsule is visible (Figure 8a, d, e). In living animals, a median brownish eye pigment spot of the nauplius eye would be visible anterior to the compound eye anlage (Figure 8d, e). The first antenna are bulky and their insertions lie in close proximity to each other; during continuing development they touch each other medially in front of the elongated and slightly tapering labrum (Figure 8a, b, d. e). The second antennae have further elongated; at the end of stage 9 they stretch over the second thoracopods covering them partly (Figure 8b, c). Their endo- and exopodite show the final tripartite segmentation. The mandibles have elongated and assumed a cylindrical shape (Figure 8a, b, d). They are enclosed laterally by the second antennae and medially by the labrum (Figure 8a, b). The lateral movement of the second maxillary buds results in the alignment of the first and second maxillary limb buds in a slightly tilted horizontal line (dashed white lines in Figure 8a, b; compare with white lines in Figure 7a). Segmentation continues in the thoracopods, now including the fifth thoracopod, which has lost its rectangular shape (Figure 8b, f, g). The number of the tiny setae at the posterior margin of the basal lobes of the second to fourth thoracopods has increased (Figure 8c). We did not detect any setae on the fifth thoracopod at this stage.

The broad carapace protrusion narrows, becoming a short triangular process and finally a broad elongated spine, the so-called carapace spine (Figure 8h-j); however, there is variation in the extent to which the spine narrows and elongates during stage 9. The carapace changes its overall shape due to indentations and tissue movement that appear on either side of the insertion of the carapace spine, and aligns with the meanwhile more pear-like shape of the embryo. The dorsal organ appears as a prominent structure in the antero-dorsal region of the carapace (Figure 8h-j). Nuclear staining indicates that the dorsal organ only contains few cells; the cells are much bigger than the ones of the surrounding tissue and only show weak nuclear staining. In crustaceans, the osmoregulatory function of the dorsal organ is well known [25].

The tissue surrounding the proctodeum protrudes due to the continuing outgrowth of the abdomen. Anterior to the proctodeum, two furcal claws [26] pointing in posterior direction are evident (arrow heads in Figure 8f). Posterior to the proctodeum the two setae described in stage 7.5 have further elongated (arrowheads in Figure 8h, j). The embryonic cuticle is now clearly visible in SEM preparations (white arrows in Figure 8h, j).

### S10: Hook-shaped abdomen

The main character of this stage is the change in shape of the abdomen due to continuous growth and a shift of the proctodeum towards anterior, which results in a hook-like shape of the abdomen.

**Figure 9:**
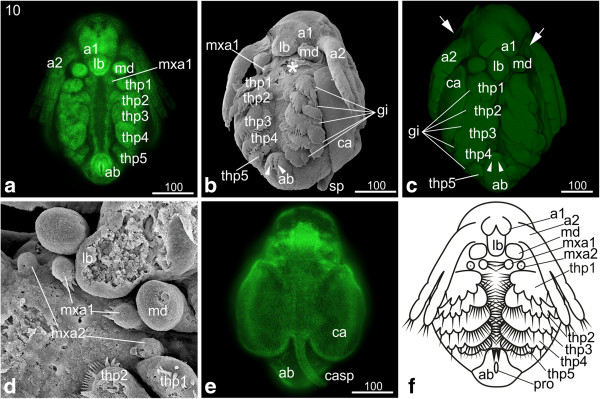
**Stage 10 (Hook-shaped abdomen): (a, e) Sytox; (b, d) SEM; (c) Congo-red; (f) schematic drawing. (a)** Ventral view: the second antennae have further elongated and stretch partly over the third thoracopod. **(b, c)** Ventro-lateral view: the abdomen appears hook-shaped due to continuous growth. The two furcal claws anterior of the proctodeum (arrowheads) have elongated and still point posteriorly. The overall shape of the thoracopods did not change, but the setae are more prominent. The fifth thoracopod just bears few tiny setae. The gills on each thoracopod have increased in size (white lines). In figure c, an egg membrane enclosing the entire embryo is evident (arrows). **(d)** The first maxillae have developed three prominent setae pointing medially. The second maxillae appear as small knobs showing the opening of the maxillary glands. **(e)** The carapace spine has developed into a narrow elongated spine, and the two halves of the carapace have assumed a seashell-like shape. **(f)** Schematic drawing of stage 10. a1, a2 = first and second antennae; ab = abdomen; ca = carapace; casp = carapace spine; gi = gill; lb = labrum; md = mandible; mxa = maxillary appendage; pro = proctodeum; thp = thoracopod.

The first antennae have not changed except for further elongation. The second antennae have also continued to elongate and reach the third thoracopod (Figure 9a, b, c). The first maxillae have developed three prominent setae (Figure 9d). The second maxillae appear as small knobs posterior-laterally to the first maxillae; they show the tiny openings of the maxillary glands (Figure 9d). The overall shape of the thoracopods did not change; they show the same segmentation as in the previous stage. However, the setae become more prominent (Figure 9b). The fifth thoracopod is not as prominently developed as the remaining ones, although tiny setae can be detected. It is subdivided into one basal segment, an epipodite (gill), an outer exopodite and an inner endite, bearing an endopodite. The size of the developing gills has increased on each leg (Figure 9b, c). The carapace spine has developed into a narrow elongated spine, and the two halves of the carapace have assumed a seashell-like shape (Figure 9e). The abdomen continues to grow leading to a further shift of the proctodeum towards anterior; the entire abdomen is situated posterior-medial to the fifth thoracopods (Figure 9a, b, c, f). The two furcal claws located anterior to the proctodeum have become much more prominent and are still pointing in posterior direction (data not shown). The embryonic cuticle now encloses the entire embryo, and the positive Congo-red staining indicates that the cuticle contains chitin (Figure 9c).

### S11: Carapace partially covers the legs

**Figure 10:**
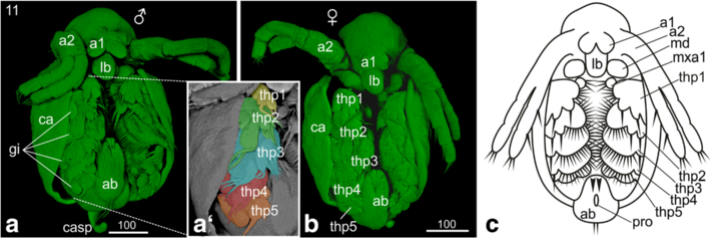
**Stage 11 (Carapace partly covers the legs): (a, b) Sytox; (a‘) Detail of thoracopods artificially colorized; (c) Schematic drawing. (a)** Male, ventro-lateral view: the seashell-like halves of the carapace have further extended and cover approximately four fifths of the dorsal side of the embryo, while on the ventral side the legs are partially covered. The bases of the first antennae adjoin each other medially. On each tip two setae are evident. The second antennae elongated and reach the fourth thoracopod. The thoracopods have developed prominent setae. The gills in the lateral area of the thoracopods have increased in size. Due to the growth of the abdomen the proctodeum has shifted even more in anterior direction. **(a‘)** The second to fourth thoracopods developed prominent setae. A few setae are evident on the fifth thoracopod. **(b)** Female, ventral view: beside the fused first antennae the females show the same habitus as the males. **(c)** Schematic drawing of stage 11. a1, a2 = first and second antennae; ab = abdomen; ca = carapace; casp = carapace spine; gi = gill; lb = labrum; md = mandible; ml = midline; mxa = maxillary appendage; pro = proctodeum; thp = thoracopod.

The labrum has further elongated and slightly narrowed (Figure 10a, b). The fusion of the first antennae progresses, but we observe differences concerning the grade of fusion which seems to be sex-related: the males’ first antennae do not fuse (only partial fusion of the most proximal part can be observed) and become much more prominent than in females during further development; they bear the aesthetascs which are olfactory organs (Figure 10a). The females’ first antennae will fuse completely except for the most distal area; in stage 11 they fuse partly (Figure 10b). Also the females’ first antennae bear setae; however, their function has not yet been demonstrated. The first signs of the developing setae can be detected at the tips of the first antennae. The second antennae have further elongated and reach the fourth thoracopod (Figure 10a, b, c). The size of the maxillae has decreased. The thoracopods have further differentiated and developed prominent setae; however, their overall shape did not change compared to the previous stage (Figure 10a, a‘, b). A few setae are evident on the fifth thoracopod (Figure 10a’). The further increasing gills are evident on the lateral area of each thoracopod (Figure 10a, a‘). The growth of the abdomen has continued causing a further shift of the proctodeum in anterior direction (Figure 10a, b). The two furcal claws anterior to the proctodeum have continued elongation (Figure 10a, a‘, b). The seashell-shaped halves of the carapace extend further posteriorly covering approximately four fifth of the dorsal side of the embryo; for the first time the legs are also partially covered. The carapace spine has further elongated and bends along the abdomen (Figure 10a).

### S12: Protruding first antennae

**Figure 11:**
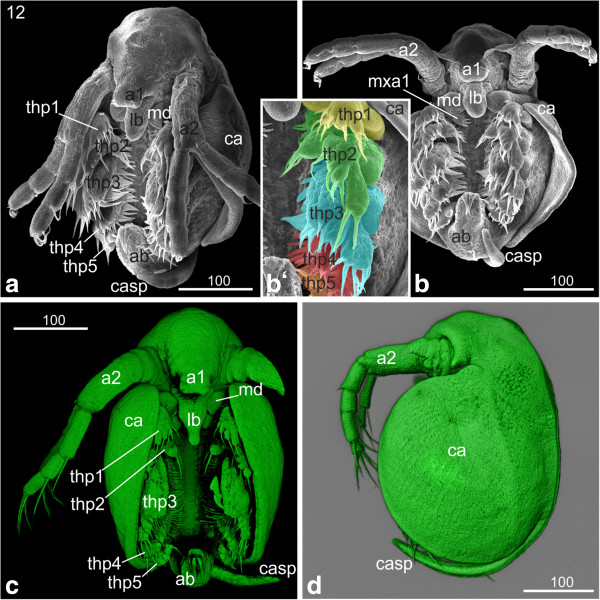
**Stage 12 (Protruding first antennae): (a-b) SEM; (c, d) Congo-red. (a)** Ventro-lateral view: the overall shape of the embryo changed to a crookbacked shape due to modifications of the anterior carapace. The head is now covered by the carapace and the first antennae are protruding. In females, the first antenna fuses completely at this stage. The second antennae are almost as long as the whole body. The labrum has continued its elongation and narrowing of the tip. **(b)** The setae and setae on the first to fourth thoracopod have enlarged. The number and size of the setae on the fifth thoracopod have increased. The proctodeum moved further anterior due to the growth of the abdomen. The carapace spine has further elongated and bends in antero-ventral direction. **(b‘)** Detail of the thoracopods showing the enlargement of the setae. **(c)** Ventral view. **(d)** The lateral view shows the change in overall habitus leading to the typical crookbacked *Daphnia*-habitus. The carapace spine elongated and bends in antero-ventral direction. a1, a2 = first and second antennae; ab = abdomen; ca = carapace; casp = carapace spine; gi = gill; lb = labrum; md = mandible; mxa = maxillary appendage; pro = proctodeum; thp = thoracopod.

The labrum has continued its elongation and narrowed at the tip (Figure 11a, b, c). More and more the embryo assumes a crookbacked shape caused by the modification of the anterior carapace; this results in the head being covered by the anterior part of the carapace (Figure 11b, c, d). Due to this modification, the first antennae, which in female embryos fuse completely during stage 12, are protruding (Figure 11a, b, c). This is also true for males who do not show fused antennae. Two setae have developed at the tip of the first antennae (Figure 11a, b, c). The second antennae continue elongation and become almost as long as the whole body (Figure 11a, c). The thoracopods have enlarged, including their setae; the number of setae on the fifth thoracopod has increased (Figure 11a, b, c).

The abdomen has continued to grow, shifting the proctodeum further in anterior direction (Figure 11a, b). The two furcal claws antero-lateral to the proctodeum have increased in size, and the two posterior setae have elongated significantly. The carapace covers now almost the entire embryo (Figure 11b, c). The carapace spine further elongates and bends in antero-ventral direction but it does still not reach the posterior end of the abdomen (Figure 11a, b, d).

#### *Hatchling and first instar*

**Figure 12:**
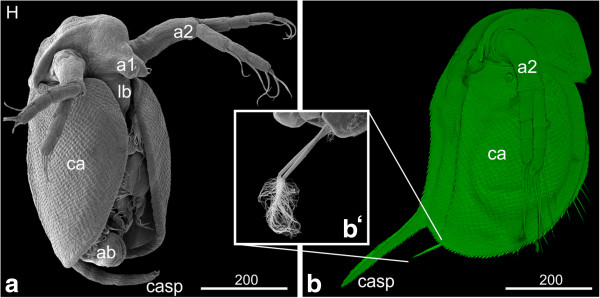
**Hatchling and first instar: (a, b‘) SEM; (b) Congo-red staining. (a)** Hatchling: the embryo has a compact form, and the seashell-like halves of the carapace cover the entire legs. The surface of the carapace shows a rhombic pattern. **(b)** First instar: the entire rim of the carapace is covered with setae. The carapace spine has stretched out and points now in dorso-posterior direction; its edges are covered with setae. **(b‘)** The two sensory setae located posterior to the proctodeum stick out almost parallel to the carapace spine. They have developed a feather-duster-like shape: at the distal part of the setae numerous curly hair-like setae are evident. a1, a2 = first and second antennae; ab = abdomen; ca = carapace; casp = carapace spine; lb = labrum.

The hatchling has a compact form, and the seashell-like halves of the carapace cover the entire legs (Figure 12a). The carapace spine still bends anteriorly. After being released into the environment, a molt takes place and the first instar starts swimming. The carapace spine has stretched out and points now in posterior direction (Figure 12b). The spine is longer than the abdomen and its margins are covered with setae (Figure 12b). The two sensory setae located posterior to the proctodeum stick out almost parallel to the carapace spine. They have developed a feather-duster-like shape: at the distal part of the setae numerous hair-like setulae are evident (Figure 12b‘). The entire rim of the carapace is covered with setae (Figure 12b). The surface of the carapace shows a rhombic pattern (Figure 12b). The compound eyes are not completely fused yet (data not shown). The hatchling still contains a lot of yolk. The gills have assumed a pouch-like shape. The thoracopods show a large number and variety of long hairs, setae and other structures.

## Discussion

### Comparative aspects of *D. magna* development and the staging system

**Figure 13:**
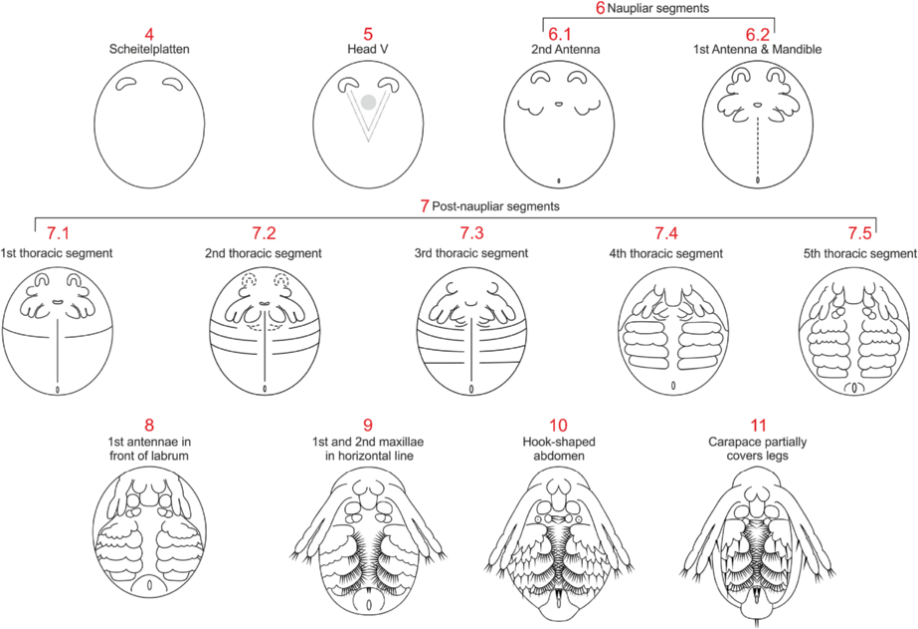
**Summary of all developmental stages of *Daphnia magna*.** The major landmarks are summarized and schemes shown where appropriate. See text for details.

Here we present for the first time a staging system for *Daphnia magna* based on morphological landmarks (Figure 13). The staging can most likely be applied to apomictic and resting eggs since it was shown in *D. pulex* that the sequence and morphology of development are the same for both types of eggs except that development is arrested in the latter in the first phase of gastrulation and resumed after a diapause [27]. Furthermore, sexual dimorphism can only be observed in late developmental stages (stage 11) and does not affected the landmarks used for staging so that the staging system can be applied to both sexes.

Until now, there was no staging system available for *Daphnia magna*, the species that is most frequently used for ecological and recently also for developmental research. Though it should be mentioned here that a few perfunctory descriptions of developmental stages in *D. pulex, D. magna* and an additional cladoceran, *Dunhevedia crassa,* have been published - some recently - which are based on hours of development [5,11,14,15]; however, these cannot be used as staging systems due to large gaps and lack of morphological landmarks.

Kotov and Boikova [13] present a staging system for D. *hyalina* and *D. galeata*, which is again based on hours of development after the eggs have been deposited into the brood pouch. The authors mainly present schematic drawings but give detailed descriptions of the stages based on the analysis of SEMs. Comparison of these descriptions to the *D. magna* staging system reveals a high correspondence in the sequence of major morphological events (Figure 13). Kotov and Boikova [13] did not include the early stages of development (0 to 10 hours in *D. hyalina* and *D. galeata*) into their staging system. The staging starts with the formation of the naupliar segments followed by the generation of the postnaupliar segments, which corresponds to the morphological landmarks of stage 6 and 7, respectively, in *D. magna*. However, the formation of the fifth (and last) thoracic segment seems to be delayed compared to *D. magna*. D. *hyalina* and *D. galeata* embryos are elongated when the fifth thoracic segment forms, while *D. magna* embryos have still a roundish appearance and only elongate after formation of the last thoracic segment. Additional landmarks such as the medial movement, fusion and protrusion of the first antennae, the lateral movement of the second maxillae, the formation of the caudal spine and sensory setae all appear in the same sequence in all three *Daphnia* species. The only other slightly heterochronic event is the ventral flexure of the abdomen which in *D. magna* occurs in stage 10 (‘hook-shaped abdomen’), while it occurs later relative to other morphological landmarks (for example, fusion of the bases of the first antennae, lateral bend of the caudal spine) in *D. hyalina* and *D. galeata.* Furthermore, we found that the length of the second antennae, which is used frequently in the Kotov and Boikova [13] staging system, is not a reliable landmark in *D. magna* since it varies greatly in relation to other morphological landmarks.

Although until now, there was no staging system available for the early stages of *Daphnia* development, several publications from the late nineteenth to the early twentieth century give very detailed descriptions of the cleavage divisions, blastoderm formation and gastrulation in various cladoceran species based on light microscopic sections [21-24,27-30]. Comparison of early stages between several cladoceran species (*D. magna*, *D. pulex*, *D. longispina*, *Moina rectirostris*, *Holopedium gibberum* and *Polyphemus pediculus*) revealed that early development is highly conserved in cladocerans and species-specific differences must occur in later development. Our data confirm that the sequence of events in early *D. magna* development is conserved, including the unequal divisions of the blastoderm cells, the formation of the ‘Scheitelplatten’, the formation and position of the gastrulation zone and the immigration of presumptive germ line cells, thus supporting previous results.

Furthermore, Fritsch and Richter [31] have compared the development of two branchiopod representatives, *Leptestheriada halacensis* (Spinicaudata) and *Cyclestheria hislopi* (Cyclestherida), which belong to taxa closely related to Cladocera. It seems that the sequence of appearance of morphological landmarks, such as the relative time of trunk segment formation, appendage differentiation and eye formation shows heterochronic changes when compared between these two species but also compared to *D. magna*. However, the morphological landmarks used by us and by Fritsch and Richter [31] are not sufficiently overlapping for a detailed comparison.

### Formation of the brain and the visual system

The ‘Scheitelplatten’ are one of the most prominent morphological landmarks of early development in Cladocera. They allow for an early determination of the anterior-posterior and dorso-ventral axes and mark the anterior-most position of the forming germ band. The development of the ‘Scheitelplatten’ has been analyzed in great detail in *Polyphemus pediculus*[24]. The anlage arises from eight blastoderm cells, which appear in the 32-cell stage embryo. The eight cells become separated into a bilateral pair of four cells, which undergo multiple divisions. Kühnemund suggests that the ‘Scheitelplatten’ together with a few surrounding ectodermal cells form the brain, including the optic ganglion and the eye. In line with these data, we show here that *Dam atonal,* whose expression is highly conserved in the developing visual system throughout the animal kingdom, is expressed in subsets of ‘Scheitelplatten’ and surrounding ectodermal cells. Furthermore, the expression pattern of *Dam snail* suggests that the area of the Head-V together with the ‘Scheitelplatten’ and associated ectodermal cells forms the brain and the mandibular neuromere. This is in contrast to Kühnemund’s [24] assumption that only the ‘Scheitelplatten’ and the surrounding cells develop into the brain.

### Are neonates part of the embryonic development? - The switch from embryonic to post-embryonic phase

Most branchiopod crustaceans show an anamorphic (indirect) mode of development, which means that development is not completed in the embryo but the so called (ortho-) nauplius larvae hatch with only three anterior-most appendages (antenna 1, antenna 2 and mandible) and a partial set of segments. Over the course of successive larval stages, additional segments and their corresponding appendages are added [16]. This anamorphic and gradual development is assumed to be plesiomorphic for branchiopods [16,32]. However, cladocerans exhibit a different developmental strategy. They undergo epimorphic (direct) development and juveniles emerge at the end of embryonic development, which show approximately the adult morphology [33,34].

For both developmental strategies, the hatching from the egg membranes demarcates the switch from embryonic to post-embryonic development, either as a free living juvenile (direct development) or as free living larva (indirect development), for example [33,34].

Arthropods commonly have two egg membranes: the outer chorion and the inner vitelline membrane [35]. The chorion is also described as ‘first egg membrane’ in crustaceans [13], which can be shed long before hatching from the vitelline membrane. The vitelline membrane is therefore also called ‘hatching membrane’ [13]. *D. hyalina, D. galeata *[13], *D. pulex *[15] and *D. magna* embryos, for example, shed the chorion before mid-embryogenesis. In the literature, a third embryonic layer is frequently mentioned. However, this is actually the embryonic cuticle, which is formed by the hypodermal cells of the embryo itself and not involved in the process of hatching [36]. In *D. magna*, the first molt occurs shortly after hatching, while in the case of *D. hyalina*, *D. galeata* and *D. pulex* moulting and hatching seem to occur around the same time.

One peculiarity of Daphniidae and other cladoceran groups is the occurrence of ‘neonate’ embryos. According to Kotov [18], ‘neonata’ represents the terminal phase of embryogenesis; however, at this stage the embryo has already hatched and is not surrounded by egg membranes anymore. Neonate embryos are covered with a thin cuticle and not fully expanded. They are almost immobile and remain within the mother’s brood pouch with their carapace spine bent ventrally (see hatchling in Figure 12a and compare with next instar in Figure 12b). Immediately after the mother releases the neonate embryos, they undergo another molt and start their post-embryonic development [18]. Although the term is widely accepted in the branchiopod research field, it causes conceptual problems as it deviates from the generally accepted view that the time point of hatching from the egg membranes demarcates the separation between embryonic and post-embryonic development. For the sake of consistency and to avoid adding to the confusion, we suggest keeping the term 'neonate' for the first post-embryonic stage after hatching. However, we would like to stress that we do not agree with the concept of including neonates into embryonic development.

## Conclusion

We present here a staging system for *Daphnia magna* which is based on morphological landmarks. The staging system can be adopted for other daphnids with minor variations since the sequence of development is highly conserved during early stages and only minor heterochronic shifts occur in late embryonic stages.

## Abbreviations

a1: first antenna; a2: second antenna; ab: abdomen; ca: carapace; cec: compound eye capsule; casp: carapace spine; do: dorsal organ; g: gut; gi: gill; gl: germ line; lb: labrum; md: mandible; ml: midline; mx1: first maxilla; mx2: second maxilla; mxa: maxillary appendage; ne: neuroectoderm; pro: proctodeum; SEM: scanning electron microscopy; sp: ‘Scheitelplatte’; sto: stomodeum; t: thoracic segment; thp: thoracopod; vne: ventral neuroectoderm.

## Competing interests

The authors declare that they have no competing interests.

## Authors’ contributions

BM, PU and CW conceived and coordinated the study. BM, PU, MK and CW did all laboratory work and analyzed the data. In addition, MK conducted the *in situ* hybridization experiments and CW carried out the 3D reconstructions. BM and PU drafted the first version of the manuscript and MK, AS and CW contributed substantially to the preparation of the final manuscript. AS and CW wrote the discussion. All authors read and approved the final manuscript.

## Supplementary Material

Additional file 1 **Part of the webpage from D Ebert’s group (Basel) dated 24.01.2014 to show details of *Daphnia *and algae culturing.** Click here for file

Additional file 2 **Expression patterns of neural genes in the ‘Scheitelplatten’ anlagen and the head-V.** Whole mounts (a-d) and flat preparations (e, f) stained with DIG-labeled RNA probes of *Dam atonal* and *Dam snail*, respectively. Anterior is towards the top. Green (Sytox), light blue (SYBR-Green), dark blue (RNA-probe). a, b: Stage 5; c, d: Stage 6; e, f: Stage 6.2. The ‘Scheitelplatte’ is surrounded by associated ectodermal cells, both anteriorly and posteriorly. The arrows point to the posterior ectodermal cells that are enclosed by the ‘Scheitelplatte’. Dam atonal is expressed in the subsets of the associated cells (arrowhead) as well as in parts of the ‘Scheitelplatten’ suggesting that these areas might contribute to the visual system. e, f: Dam snail is expressed in neuroblasts and neural precursors. The arrow points to expression in the head neuroectoderm. In addition, Dam snail is expressed in the segmental borders (arrowheads). Click here for file
