# Supplementary material for: Development and staging of the water flea Daphnia magna (Straus, 1820; Cladocera, Daphniidae) based on morphological landmarks
Source: EvoDevo. 2014 Mar 18;5:12. doi: 10.1186/2041-9139-5-12 (PMC4108089; doi:10.1186/2041-9139-5-12)
Supplement: Additional file 1 — Part of the webpage from D Ebert’s group (Basel) dated 24.01.2014 to show details of Daphnia and algae culturing. [file 2041-9139-5-12-S1.pdf]

Back to Online View

Artificial *Daphnia* medium: ADaM (Aachener Daphnien Medium)

We grow *Daphnia* in artificial medium. The original recipe for the medium was developed by Klüttgen, B., Dülmer U., Engels, M. & Ratte H. T. 1994. ADaM, an artificial freshwater for the culture of zooplankton. Water Research 28:743-746. We use a modified version of this medium (altered Seleniumdioxid concentration). To produce a given amount of medium add the amounts of sea salt and stock solutions as given in the following table.

Water	Sea salt [gram]	Stock solution A (ml)	Stock solution B (ml)	Stock solution C (ml)
10 l	3.33	23	22	1
50 l	16.6	115	110	5
60 l	19.9	138	132	6

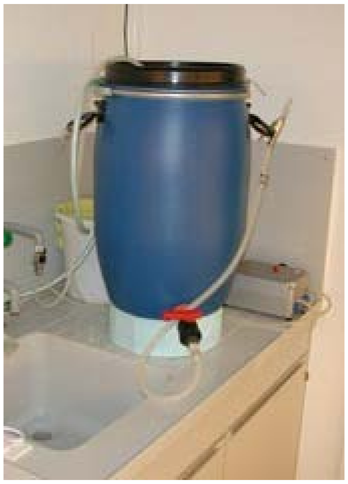

60 l medium tank in our lab

We use a special sea salt which is produced by combining all chemical to make up the mixture. (Most commercially available sea salt is produced by evaporation of water from sea water. In this case, some of the salts do not solve anymore once put back into water). We buy this sea salt from an aquarium supply company. Atrificial sea salt works fine.

These are the concentrations for the three stock solutions:

Stock solution	Chemical	Concentration [g/l]
A	$CaCl_2 \times 2H_2O$	117.6
B	$NaHCO_3$	25.2
C	SeO_2	0.07

Some notes on our experience with this medium:

- We use 60 liter plastic tanks to prepare the medium (see picture). The salts do not harm the plastic. It is better to use non-transparent material to prevent growth of algae.
- We produce the medium 24 hours in advance, but shorter time periods don't really seem to harm the *Daphnia*.
- The tanks are constantly aerated. The air passes through a filter with 0.2 µm pores. This prevents small particles and microbes to enter the water. Note that this saturated the medium with gas. If the medium warms up you get small gas bubbles in the water, which may cause the *Daphnia* to float on the water surface. If your animals have this problem allow the medium to stand still for one day before you use it.
- We keep the stock solutions in a fridge and had never problems with older solutions.
- If the tanks are used over longer period, you may get some growth of fungi and/or algae. We clean the ADaM tank regularly to keep this down.
- We have good experience with this medium culturing *Daphnia magna*, *pulex*, *longispina*, *galeata* and *hyalina*, as well as hybrids of *galeata* × *hyalina*. The inventors of the medium tested it successfully for other cladoceran species as well.
- If you have problems culturing *Daphnia* or other cladocerans in this medium, try to add some natural water (lake, stream, spring water). Adding 20% spring water we had good experience with clones which were tricky to keep otherwise.

Universität Basel, Zoologisches Institut, Evolutionsbiologie,
Vesalgasse 1, CH-4051 Basel, Switzerland

date: 24 January 2014

Back to Online View

Culturing *Daphnia* food

Please cite as: Ebert, D. Web-guide to Daphnia parasites.

We grow the green algae *Scenedesmus* in chemostats. The chemostats have a capacity of about 2.5 liters and a flowthrough of 2 liters per day. We use the following medium, which is routinely produced by mixing 7 stock solutions with TES in deionized (or destilled) water. To produce 20 liter algae medium add to 20 liter water: 2.3 g TES and 20 ml of each of the 7 stock solutions A - G (in this order!). Afterwards we pump the entire medium through a 0.2 micro-meter filter to sterilize it. (Alternative sterilize in autoclave)

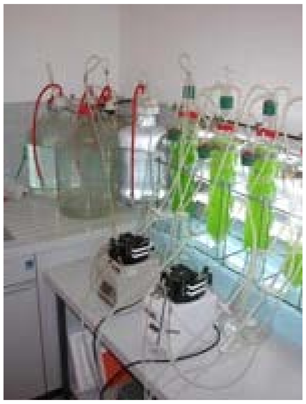

Five chemostats with peristaltic pumps in the foreground and feeding bottles in the background

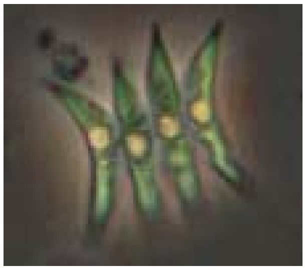

Green algae Scenedesmus

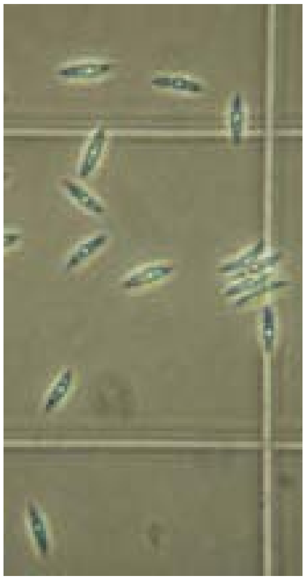

Scendesmus in a counting chamber

Stock solution	Chemical	Concentration [g/l]
A	$CaCl_2 \times 2\ H_20$	36.8
B	$MgSO_4 \times 7\ H_2O$	37.0
C	$NaHCO_3$	12.6
D	$K_2HPO_4 \times 3\ H_2O$	11.4
E	$NaNO_3$	85.0
F	$Na_2SiO_3 \times 5\ H_2O$	21.2
G*	NaEDTA	4.360
	$FeCl_3 \times 6\ H_2O$	3.150
	$CuSO_4 \times 5\ H_2O$	0.010
	$ZnSO_4 \times 7\ H_2O$	0.022
	$CoCl_2 \times 6\ H_2O$	0.010
	$MnCl_2 \times 4\ H_2O$	0.180
	$Na_2MoO_4 \times 2\ H_2O$	0.006
	H_3BO_3	1.000

** Solution G contains among others, all the trace elements.*

Some notes on how we run the chemostats:

- Change the light bulbs regularily. We change them once or twice per year. They burn out and decreae in power.
- Keep the flow rate high, other wise bacteria take over.
- We run several chemostats at a time and replace them after about 6 weeks. The algae quality decreases when the chemostats run for much longer.

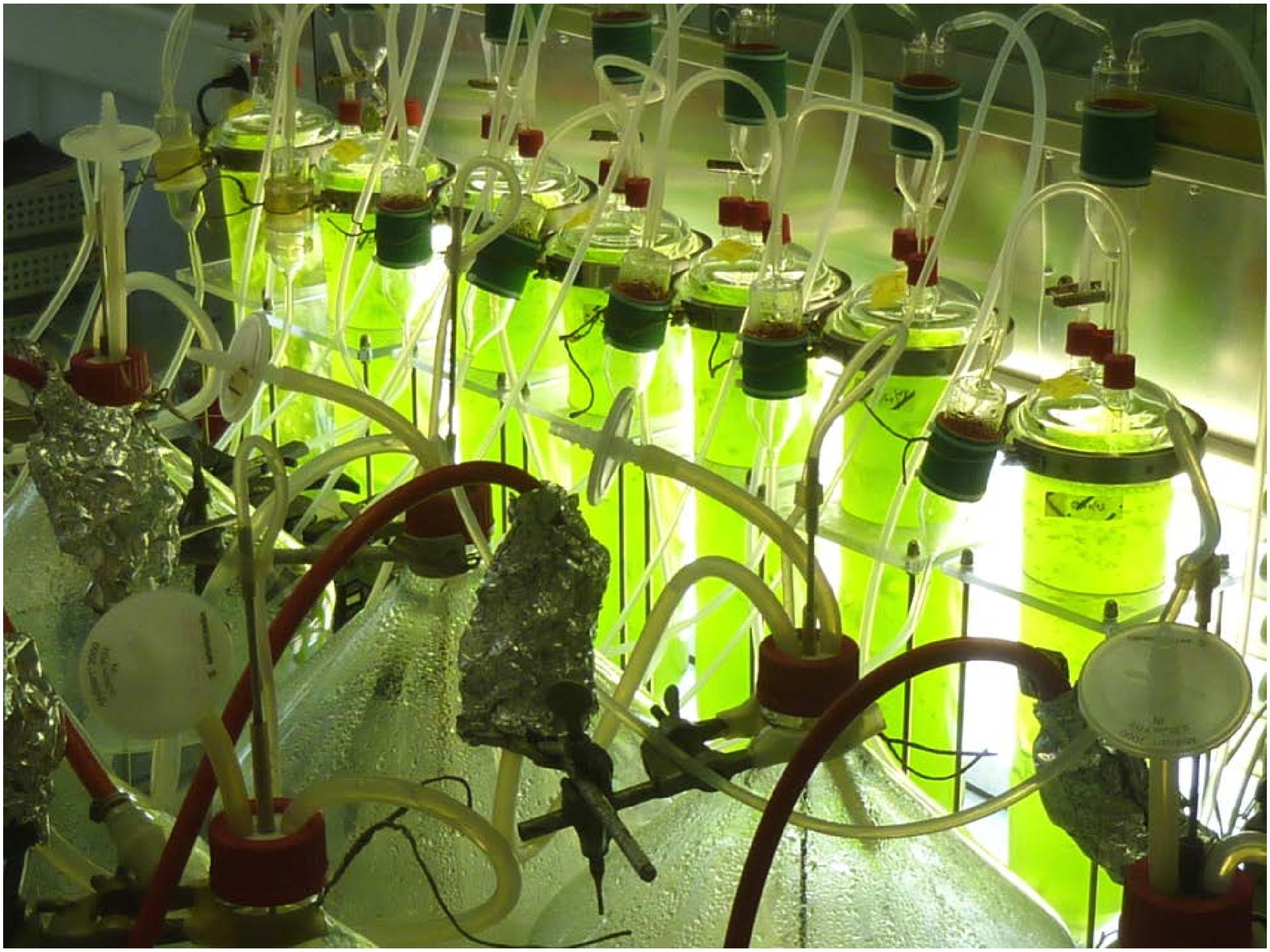

Full set of chemostats for algae mass production

Universität Basel, Zoologisches Institut, Evolutionsbiologie,
Vesalgasse 1, CH-4051 Basel, Switzerland

date: 24 January 2014
