# Supplementary figures and images for: Development and staging of the water flea Daphnia magna (Straus, 1820; Cladocera, Daphniidae) based on morphological landmarks
Source: EvoDevo. 2014 Mar 18;5:12. doi: 10.1186/2041-9139-5-12 (PMC4108089; doi:10.1186/2041-9139-5-12)

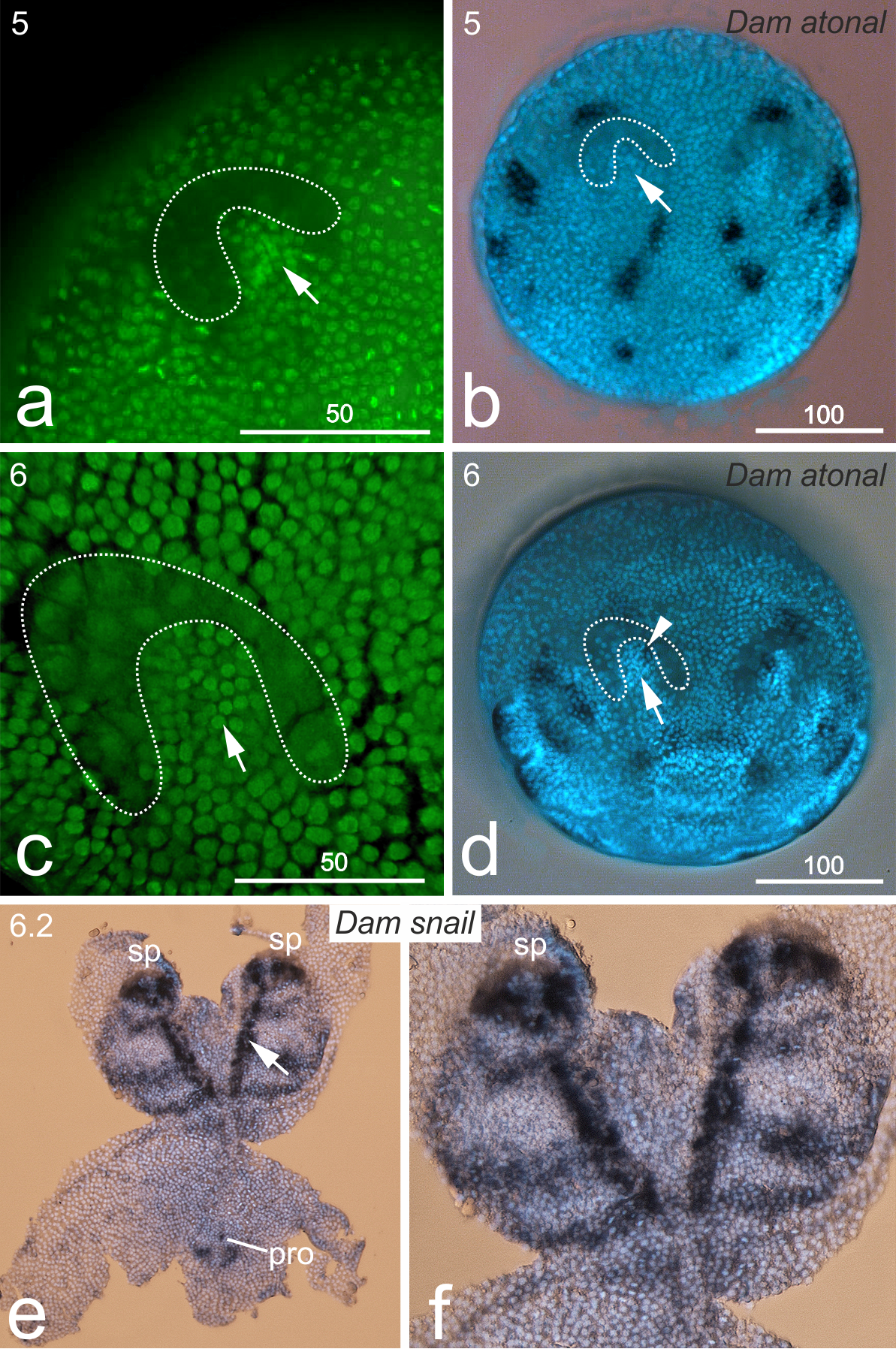

Supplement: Additional file 2 — Expression patterns of neural genes in the ‘Scheitelplatten’ anlagen and the head-V. Whole mounts (a-d) and flat preparations (e, f) stained with DIG-labeled RNA probes of Dam atonal and Dam snail, respectively. Anterior is towards the top. Green (Sytox), light blue (SYBR-Green), dark blue (RNA-probe). a, b: Stage 5; c, d: Stage 6; e, f: Stage 6.2. The ‘Scheitelplatte’ is surrounded by associated ectodermal cells, both anteriorly and posteriorly. The arrows point to the posterior ectodermal cells that are enclosed by the ‘Scheitelplatte’. Dam atonal is expressed in the subsets of the associated cells (arrowhead) as well as in parts of the ‘Scheitelplatten’ suggesting that these areas might contribute to the visual system. e, f: Dam snail is expressed in neuroblasts and neural precursors. The arrow points to expression in the head neuroectoderm. In addition, Dam snail is expressed in the segmental borders (arrowheads). [file 2041-9139-5-12-S2.png]
